# High Frequency of *AdeA, AdeB* and *AdeC* Genes among *Acinetobacter baumannii* Isolates

**DOI:** 10.18502/ijph.v49i8.3898

**Published:** 2020-08

**Authors:** Reza RANJBAR, Shahin ZAYERI, Davoud AFSHAR

**Affiliations:** 1. Molecular Biology Research Center, Systems Biology and Poisonings Institute, Baqiyatallah University of Medical Sciences, Tehran, Iran; 2. Department of Microbiology and Virology, School of Medicine, Zanjan University of Medical Sciences, Zanjan, Iran

**Keywords:** *Acinetobacter baumannii*, Antibiotic resistance, Gene

## Abstract

**Background:**

Efflux pumps are involved in resistance of *Acinetobacter baumannii* isolates to antimicrobial agents. AdeABC efflux pump is one of the RND superfamily efflux pump and consists of *adeA* (membrane fusion), *adeB* (multidrug transporter) and *adeC* (outer membrane) genes. In this study, the frequency of *adeA, adeB* and *adeC* genes among *A. baumannii* isolates with resistance to erythromycin, trimethoprim, meropenem and imipenem was investigated.

**Methods:**

Overall, 79 strains of *A. baumannii* were isolated from patients admitted to two major hospitals in Tehran during 2016. Antibiotic susceptibility testing was determined by disc diffusion and microdilution methods according to Clinical and Laboratory Standards Institute (CLSI) guideline. The presence of *adeA, adeB* and *adeC* genes was also determined using Multiplex PCR assay.

**Results:**

The highest and the lowest resistance among *A. baumannii* isolates were to trimethoprim (93%) and erythromycin (53%), respectively. The frequency of *adeA, adeB* and *adeC* genes was 96.2%, 96.2% and 91.1 % respectively. There was a significant relationship between imipenem resistance and presence of efflux pump genes (*P*<0.05).

**Conclusion:**

According to the high prevalence of the AdeABC efflux system genes, it may involve in resistance of clinical isolates of *A. baumannii* to imipenem, especially.

## Introduction

Bacteria belong to genus *Acinetobacter* are important pathogenic agents of human diseases (1). *Acinetobacter baumannii* is known as the most frequently isolated species from human’s infection (2–5). The species is widely distributed in environments and may be found in soil, water and sewage, as well as in variety of foodstuffs (6).

Hospital environment, especially intensive care units (ICU) are the most important sites of their occurrence (7–9). As the second most commonly isolated nonfermenters bacteria in human specimens, *A. baumannii* is inhabitants of healthy human skin. Other common reservoirs of these organisms include moist and dry surfaces, where they can easily survive for many days or weeks (10). On the other hand, *A. baumannii* has been the main cause of all nosocomial infections because of its ability to acquire resistance determinants and biofilm formation on surfaces, which allow them to become resistant to a wide range of antibiotics (11).

*A. baumannii* causes many infections including bloodstream infections, ventilator-associated pneumonia (VAP), urinary tract infections (UTI) and wound infections (12). The risk factors of *A. baumannii* infections are instrumentation, mechanical ventilation, surgery, treatment with broad-spectrum antibiotics, and hospitalization at ICU (13).

Due to the vast consumption of antibiotics and the extension of *A. baumannii* multi-drug resistant (MDR) strains, its morbidity and mortality has increased in last decade. (14). The MDR *A. baumannii* strains often are susceptible only to a few antibiotic classes (carbapenems and polymyxins) and resistance to other antimicrobial classes (15). Colonization of strains in the gastrointestinal tract in hospitalized patients at ICU is an important epidemiologic reservoir for *A. baumannii* (MDR strains) infections in hospital outbreaks (16). In the last few decades *A. baumannii* has emerged as important opportunistic pathogen, especially as multiple resistant to the major antimicrobial agents which used to treat nosocomial infections.

Efflux pumps genes, β-lactamases, integrons, and insertion sequence (IS) elements are considered as the most prevalent determinants among MDR *A. baumannii* strains (17). Bacterial efflux pumps are complicated in clinically related resistance to antibiotics, bacterial colonization and the persistence of bacteria in the host (18). Chromosomally-encoded pump is tripartite efflux machinery belongs to the RND-type superfamily. The AdeABC efflux pump consists of *adeA* (membrane fusion), *adeB* (multidrug transporter) and *adeC* (outer membrane protein) genes. These genes are contiguous and adjacent by two-component regulatory systems; *adeR* and *adeS* (19).

The major efflux mechanism associated with carbapenems resistance in *A. baumannii* is the chromosomally-encoded tripartite efflux pump, AdeABC, found in approximately 80% of clinical strains of *A. baumannii*. Inappropriate antibiotic therapy can induce more expression AdeABC efflux pump. Hence, the over-expression of AdeABC efflux pump confers resistance to aminoglycosides and decreases susceptibility to fluoroquinolones, tetracycline, chloramphenicol, erythromycin, trimethoprim and ethidium bromide, netilmicin and meropenem (20). The synergy between acquired oxacillinases and the AdeABC pump has been implicated in the higher levels of resistance to β-lactams.

We aimed to investigate the frequency of *adeABC* genes and antibiotic resistance to erythromycin, trimethoprim, meropenem and imipenem among *A. baumannii* strains, from two hospitals in Tehran, Iran.

## Materials and Methods

### Bacterial isolation and antimicrobial susceptibility testing

During 85 days since May 4th 2016, overall 79 clinical strains of *A. baumannii* were subjected to the study. These isolates have been recovered previously from patients in two major hospitals (Baqiyatallah and Tehran Heart Hospitals) in Tehran, Iran. The clinical strains were cultured in triple sugar iron agar (TSI), sulfide-indole-motility (SIM), methyl red and Voges-Proskauer (MR-VP) test and identified according to the conventional biochemical tests, API20 E and Vitek2 systems (BioMerieux, Marcy-l’Etoile, France). Antimicrobial susceptibility test was also performed using disc diffusion method according to Clinical and Laboratory Standards Institute guidelines (CLSI).

### Detection of AdeABC efflux pump system by PCR

*A. baumannii* strains were assayed for presence *ade*ABC genes by PCR method with the specific primers showed in Table 1. DNA extraction was carried out using a procedure as described by Hassanzadeh, et al. (21). The condition for amplification of *ade*ABC genes was as follows: initial denaturation at 94 °C for 5 min, 30 cycles of 94 °C for 1 min, 55°C for 1 min, 72 °C for 1 min and a final amplification at 72 °C for 5 min. Amplicons were electrophoresed in gel agarose 1%, stained with ethidium bromide and visualized under UV transilluminator documentation system (Bio-Rad).

**Table 1:** The primers used in study

***Primer name***	***Sequence (5′ →3′)***	***Amplicon size***
Ade.A	F: GAAATCCGTCCGCAAGTC R: ACACGCACATACATACCC	683 bp
Ade.B	F: AAAGACTTCAAAGAGCGG R: TCACGCATTGCTTCACCC	623 bp
Ade.C	F: ATTTCAGGTCGTAGCATT R: CTTGATAAGTAGAGTAGGGATT	370 bp

### Statistical Analysis

All data were analyzed using conducting Chi-square method using SPSS software, version 16 (Chicago, IL, USA). The *P* value less than 0.05 was used as the cutoff for significance.

## Results

**Fig. 1:**
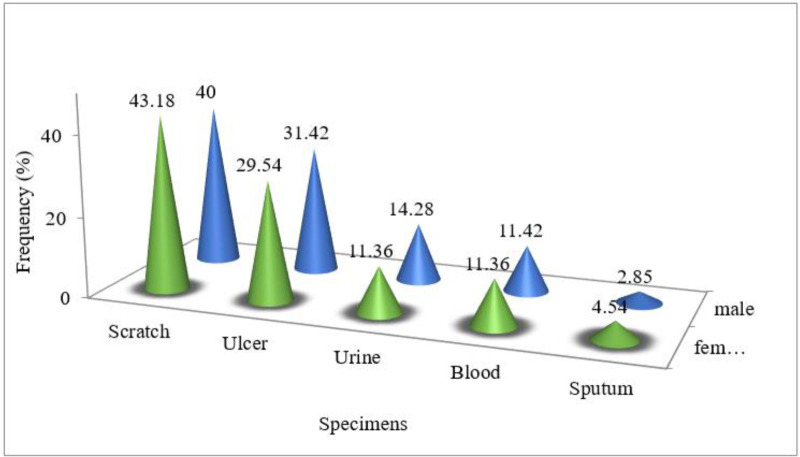
The frequency of *A. baumannii* strains isolated from the clinical specimens.

**Fig. 2:**
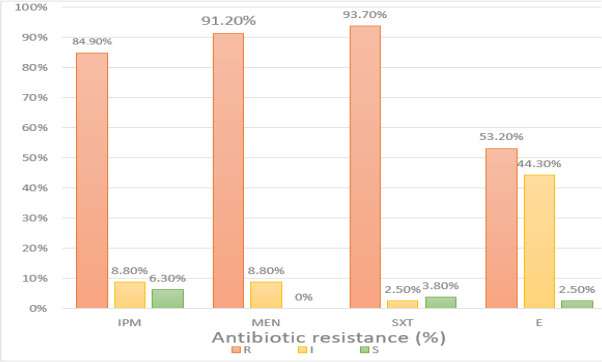
Antibiotic resistance of *A. baumannii* isolates

From 79 strains of *A. baumannii* isolated from clinical specimens, 35 isolates obtained from males (44%) and 44 strains belonged to females (56%). In fact, in terms of gender, the prevalence of *A. baumannii* strains was almost the same in females as males. The strains in this study were obtained from scratch, ulcer, urine, blood and sputum sources. The frequency of bacteria extracted from the specimens is shown Fig. 1. The results of antibiotics susceptibility test showed that the highest and the lowest resistance of *A. baumannii* were to trimethoprim (93.7%) and erythromycin (53.2%), respectively (Fig. 2).

**Fig. 3:**
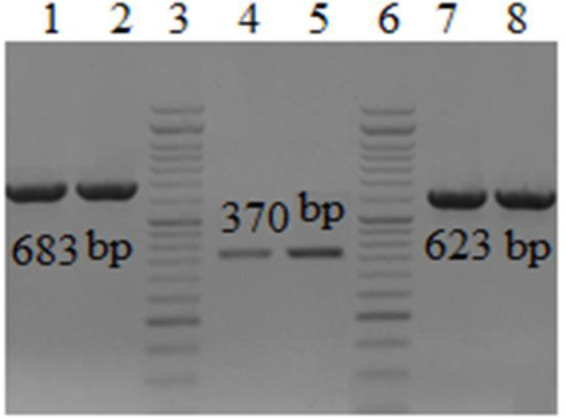
Electrophoresis of *adeA, adeB* and *adeC* amplicons of three selected isolates of *A. baumannii* in gel agarose 1%. Lanes 1–2, PCR product of *adeA* gene (683 bp); lanes 3&6, DNA ladder 100-bp; lane 4–5, PCR product of *adeB* gene (370 bp). Lanes 7–8, PCR product of *adeC* gene (623 bp)

**Fig. 4:**
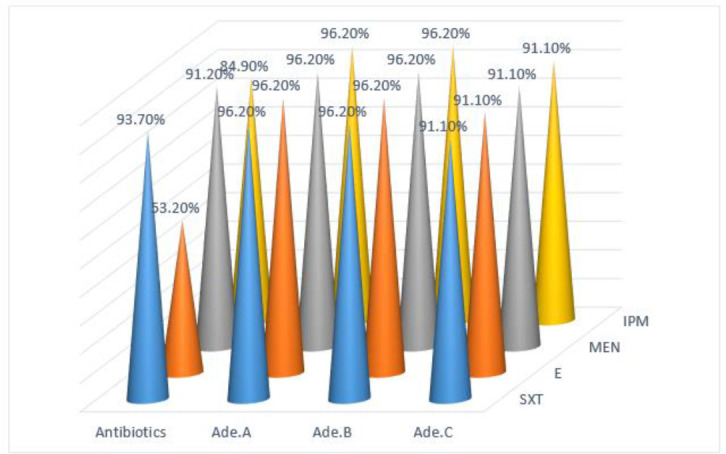
The rate of efflux pump existence in the drug resistant phenotypes in *A. baumannii* isolates

Genes encoding efflux pumps in all 79 MDR isolates were determined by multiplex-PCR assay. Amplicons of *adeA*, a*deB* and a*deC* genes were 683bp, 623 bp and 370 bp, respectively (Fig. 3). From a total of 79 isolates, the percent of isolates with *adeC* gene were 91.1% (high significant differences at *P* < 0.01). The effect of Phe-Arg-Beta-Naphthylamide (PAβN) on MICs of *A. baumannii* is summarized in Fig. 4. There was no statistically significant difference between the erythromycin resistance and presence of *adeA, adeB, adeC* genes (chi-square test), while a significant difference was observed between the resistance to trimethoprim and *ade*C gene (chi-square test, *P* <0.05).

## Discussion

The novelty of the current study was to detect and determine the prevalence of efflux pump genes in MDR strains of *A. baumannii* isolated from Tehran, Iran. A total of 56 multidrug resistant isolates of *A. baumannii*, the *adeA, adeB* and *adeC* genes detected in 100%, 100% and 96.5% of isolates, respectively. Furthermore, all isolates were resistant to cefotaxime, ceftazidime, cefepime, cefoxitin, azteronam, ciprofloxacin and imipenem.

The results revealed that all of *A. baumannii* strains isolated from burn units were positive for *adeA* and *adeB* genes, while *adeC* gene was found in 85% of isolates (22). The most of carbapenems resistant *Acinetobacter* spp. was positive for *adeA, adeB, adeR, adeS* and *adeC* genes (23).

It is considered the frequency of these resistance genes to be high, so that in China, 88.2% of multidrug resistant isolates of *A. baumannii* isolated from hospitals carried genes of AdeABC efflux system (24). The majority of imipenem resistant *A. baumannii* isolates (> 80%) was positive for *adeB, adeR, adeS, adeJ* and *abeM* genes (25).

The *adeB* efflux pump gene existed in all MDR *A. baumannii* strains isolated from five hospitals in Taiwan (26). Insertional inactivation of *adeB* in *A. baumannii* strain BM4454 showed that it is involved in resistance against many antimicrobial agents including aminoglycoside, tetracycline, fluoroquinolones, erythromycin, trimethoprim and ethidium bromide (27). Additionally, the overexpression of AdeABC system and mutations in the *adeR/S* genes (encoding a two-component regulatory system) constitutes a major mechanism of multidrug resistance in nosocomial strains of *A. baumannii* (28).

Our results showed that the most of multidrug resistant isolates possessed the all *adeA, adeB* and *adeC* genes but it was obvious that the *adeB* and *adeA* genes had the main role in the resistance mechanism.

The effects of efflux pump inhibitors such as carbonyl cyanide 3-chlorophenylhydrazone (CCCP) and Phe-Arg β-naphthylamide (PAβN) on susceptibility to antimicrobial agents has been investigated, previously (29). The addition of the PAβN at different concentrations reduced the MICs of different antibiotics such as ertapenem (30).

Statistical analysis showed no significant relationship between the resistance to erythromycin and the presence of *adeABC* genes but in the case of three other antibiotics, there was a significant relationship between antibiotic c resistance and the presence of *adeABC* genes. It can be concluded that the presence of *adeABC* genes can stimulate the resistance to imipenem and trimethoprim in *A. baumannii* strains. Our results also suggested that drug efflux pumps contribute to the resistance to carbapenem in *A. baumannii* clinical isolates.

Our results are concordant with the findings that believed the *adeC* gene is not essential for *A. baumannii* MDR phenotypes (19). Overexpression of OXA-23 carbapenemase and AdeABC efflux pump may contribute to carbapenem resistance in clinical isolates of *A. baumannii* (31). Our results disagree with the data of some previous studies which indicated the AdeABC efflux pump were present in the both carbapenem-resistant and sensitive strains, therefore they might do not involve in *A. baumannii* carbapenems resistance (32, 33).

## Conclusion

The current study demonstrated the main contribution of the *adeB* gene and its regulatory system in multidrug and carbapenems resistance in clinical isolates of *A. baumannii*.

## Ethical considerations

Ethical issues (Including plagiarism, informed consent, misconduct, data fabrication and/or falsification, double publication and/or submission, redundancy, etc.) have been completely observed by the authors.
